# Unified genomic and chemical representations enable bidirectional biosynthetic gene cluster and natural product retrieval

**DOI:** 10.1038/s41598-026-49955-5

**Published:** 2026-05-09

**Authors:** Guimei Liu, Yiting Li, Gabriel Ong, Fong Tian Wong, Dillon W. P. Tay, Yee Hwee Lim, Chuan Sheng Foo, Winston Koh

**Affiliations:** 1 Institute for Infocomm Research (I2R), Agency for Science, Technology and Research (A*STAR), 1 Fusionopolis Way, #21-01 Connexis, Singapore, 138632 Singapore; 2 Institute of Sustainability for Chemicals, Energy and Environment (ISCE2), Agency for Science, Technology and Research (A*STAR), 8 Biomedical Grove, #07-01 Neuros Building, Singapore, 138665 Singapore; 3 Institute of Molecular and Cell Biology (IMCB), Agency for Science, Technology and Research (A*STAR), 61 Biopolis Drive, #07-06, Proteos, Singapore, 138673 Singapore; 4 Bioinformatics Institute (BII), Agency for Science, Technology and Research (A*STAR), 30 Biopolis Street, #07-01, Matrix, Singapore, 138671 Singapore

**Keywords:** Biotechnology, Computational biology and bioinformatics

## Abstract

Natural product discovery is increasingly driven by the ability to analyze microbial genomes for biosynthetic gene clusters (BGCs) that encode secondary metabolites. While existing approaches have successfully linked BGCs to broad classes of chemical products, they typically operate in a single modality (genomic or chemical) limiting the scope of bidirectional prediction. In this work, we propose a multimodal framework that integrates genomic and chemical information by projecting embeddings derived from pretrained language models into a common representation space. We embed genomic sequences using a BGC foundation model and represent molecules through a chemical language model, then use a metric learning model to co-embed BGCs and their associated chemical structures. This co-embedding space allows us to quantify the similarity between BGCs and compounds using similarity measures, enabling both efficient forward and inverse retrieval tasks. Our approach consistently outperforms the non-alignment approach and represents a generalizable, scalable strategy to bridge biological and chemical modalities in natural product discovery.

## Introduction

The discovery of natural products increasingly relies on the sequencing of genomes to annotate and identify biosynthetic gene clusters (BGC) that produce bioactive compounds^1,2^. Assigning a BGC sequence to its chemical product class is currently done by comparing sequence homology and domain architecture of a novel BGC to those of known BGCs with known products using Hidden Markov Model (HMM)-based tools (e.g. antiSMASH^3^ and PRISM^4^), and more recently deep learning models (e.g. DeepBGC^5^ and BiGCARP^6^). This has been very effective for annotation and broad classification of BGC sequences into major chemical classes of natural products (e.g. polyketides, saccharides, terpenes etc). However, there is an increasing need to address the inverse problem of working backwards from a target compound to select the appropriate BGC(s) capable of producing it or similar compounds. One way to achieve this would be multimodal projection of BGC sequences and chemical structures, which are two fundamentally disparate modes of information, into a common quantitative representation space.

The integration of genomics and metabolomics has emerged as a powerful strategy for natural product discovery^7^. Existing frameworks for linking genomics to metabolomics such as NPLinker^8^ and NPClassScore^9^ provide pragmatic scoring and correlation approaches to connect spectra and gene clusters, but they operate largely by complementary scoring and correlation rather than learned cross-modal representations. Recent advances in multimodal deep learning have revolutionized cross-modal search applications, spanning domains such as text-image retrieval (e.g. CLIP^10^) and biological function prediction through natural language embeddings (e.g. ProteinCLIP^11^). At the core of these breakthroughs is the use of vector embeddings derived from deep learning models that encode diverse data types into unified numerical representations. Foundation models for BGCs, such as BiGCARP^6^ and DeepBGC^5^, alongside protein language models like ESM C^12^, chemical language models like ChemBERTa-2^13^, MoLFormer^14^, MolGPT^15^ and Uni-Mol2^16^, illustrate the potential of these embeddings by capturing the structural and functional essence of their respective domains. These embeddings have shown utility in a variety of downstream tasks, from prediction of protein solubility^17^ in bioinformatics to prediction of bioactivity in cheminformatics^14^.

**Fig. 1:**
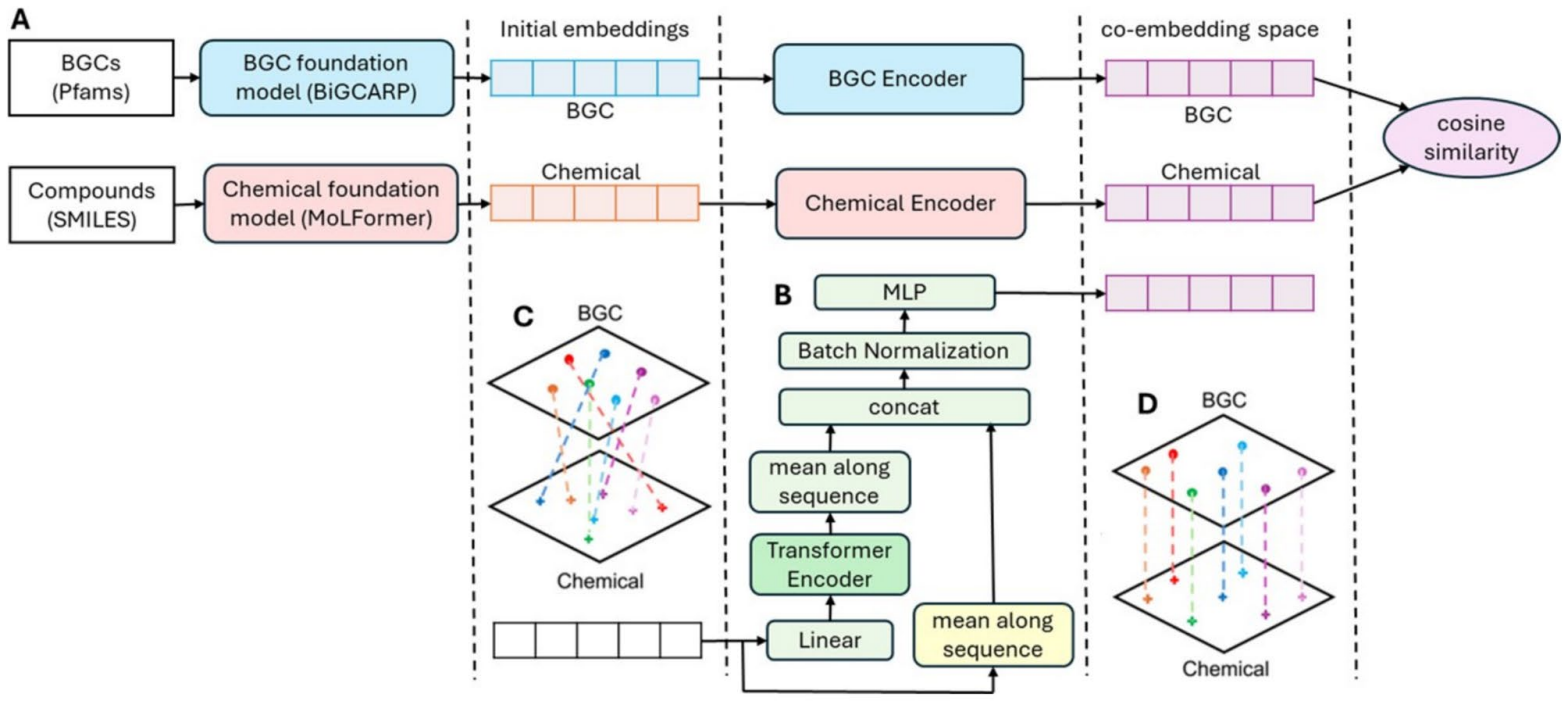
Architecture of our BCCoE model. (**A**) Overall model architecture. (**B**) Encoder architecture. BGC encoder and chemical encoder have the same architecture, but they do not share model weights. (**C**) Initial embeddings of BGCs and compounds are not aligned. (**D**) Embeddings of BGCs and compounds from same pairs are well aligned and close to each other in the co-embedding space.

Given the demonstrated richness of information captured by these foundation models, we reasoned that their embeddings, when projected in a common space, could reveal nuanced biochemical relationships between genomic and chemical modalities^18,19^. To train such a model, we came up with a cross-modal framework called BGC-Chemical Co-Embedding (BCCoE) that trains on paired examples of BGC sequences and their known small-molecule outputs (Fig. 1) from the curated MIBiG database^20^. We hypothesize that the resulting common embedding space would produce a joint representation that effectively captures the genotype-chemotype relationship underlying BGC and their natural products. Such a shared space has the potential to facilitate seamless integration and retrieval across biological and chemical domains, enabling cross-modal analyses and downstream applications. One such application identified is bidirectional retrieval. Starting with a target compound, the joint embeddings could enable the identification, ranking, and prioritization of BGCs most likely to be responsible for its biosynthesis.

A concurrent work BGC-MAP^21^ tackles the same bidirectional retrieval task as our work, but it uses a foundation model, ESM-2^22^, on BGCs only. The representations of SMILES are learned within the proposed model on compounds from MIBiG 4.0 dataset during the training process. Given the small number of compounds in MIBiG 4.0, the chemical representations learned by BGC-MAP capture much less information than the chemical foundation models used in our work. Furthermore, BGC-MAP does not map the two modalities into a common embedding space. The linking of BGCs and compounds is done through multi-head attention, which is much more costly than similarity search in a common embedding space. We compared the performance of our work with BGC-MAP on the bidirectional retrieval of three experimentally validated BGC-compounds pairs: (PX048350, dapalide A)^23^, (PV754024, lyngbyapeptin A)^24^ and (KY464182, merosterol A)^25^. For both retrieval tasks, the three pairs are ranked much higher by our BCCoE model than by BGC-MAP, illustrating the utility of our approach in sharpening both BGC and compound prioritization.

## Methods and materials

### BCCoE: BGC-chemical co-embedding for alignment

We hypothesized that projecting biosynthetic gene clusters (BGCs) and their corresponding natural products into a shared latent space could reveal underlying biochemical relationships through spatial proximity. To evaluate this hypothesis, we developed a cross-modal representation learning framework, termed BGC-Chemical Co-Embedding (BCCoE), which aligns genomic and molecular information within a unified vector space (Fig. 1). Our approach builds on recent advances in large-scale pre-trained language models, which are capable of capturing complex structural and functional representations. Specifically, we employed BiGCARP^6^ to encode Pfam domain sequences of BGCs and MoLFormer^14^ to generate embeddings of chemical SMILES strings. These deep learning models provide informative, modality-specific embeddings; however, the resulting representations initially reside in distinct and unaligned spaces, which necessitates an additional mapping strategy to bring the two modalities into correspondence.

#### Problem statement

Formally, let $\mathcal {B}$ be the space of BGCs and $\mathcal {M}$ be the space of compounds. If a compound $m\in \mathcal {M}$ is a natural product of a BGC $b\in \mathcal {B}$, then (*b*, *m*) is called a *positive pair*; otherwise (*b*, *m*) is called a *negative pair*. Let $\mathcal {D} = {(b_i, m_i)}_{i=1}^n$ be a dataset of known positive BGC-compound pairs where $b_i \in \mathcal {B}$ and $m_i \in \mathcal {M}$. The MIBiG database is one example of such a dataset. Pairs not in $\mathcal {D}$ are either unknown positive pairs or negative pairs. Our goal is to discover new unknown positive pairs by using data in $\mathcal {D}$ to learn the connections and linkage between BGCs and compounds. More specifically, we learn two functions $f_\mathcal {B}: \mathcal {B} \rightarrow \mathbb {R}^d$ and $f_\mathcal {M}: \mathcal {M} \rightarrow \mathbb {R}^d$ to map BGCs and compounds into a *d*-dimensional co-embedding space so that embeddings of a BGC and a compound from a positive pair are close to each other and embeddings of a BGC and a compound of a negative pair are farther apart. We hypothesize that BGCs and compounds from the same unknown positive pairs should be close to each other in the co-embedding space as well, and given one element of an unknown positive pair, the other element can be efficiently retrieved using nearest neighbor search in the co-embedding space.

#### Model architecture

The overall architecture of our BCCoE model is shown in Fig. 1A. BGC Pfam sequences and compound SMILES are converted to initial embeddings using a BGC foundation model and a chemical foundation model respectively. The initial embeddings of BGCs and compounds are in different embedding spaces, so they are not aligned as shown in Fig.  1C. We then use a BGC Encoder and a chemical Encoder to map the initial embeddings to embeddings in a co-embedding space so that the mapped embeddings of BGCs and compounds from same positive pairs are aligned and close to each other in the co-embedding space as shown in Fig.  1D.

**Fig. 2:**
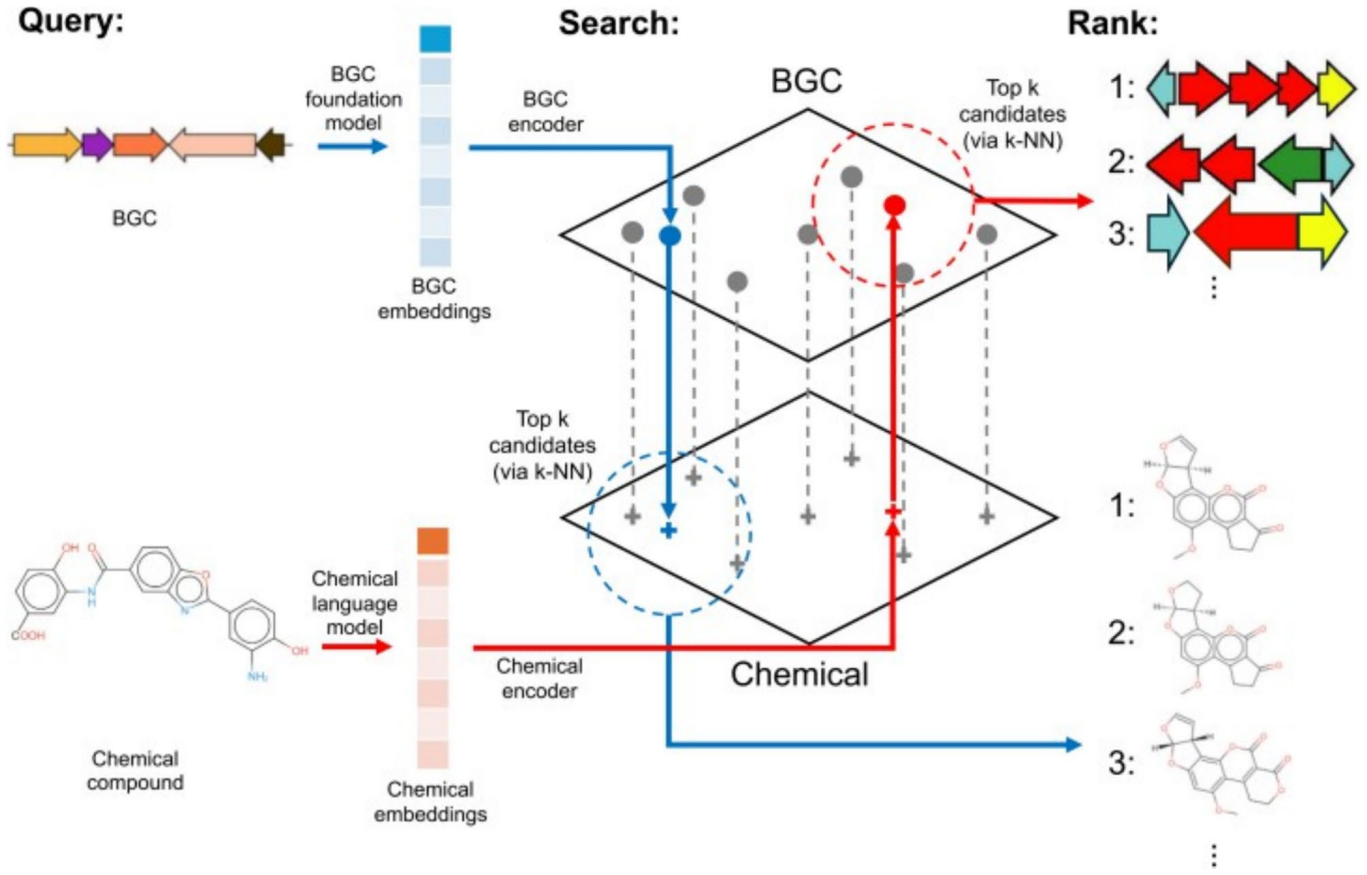
Queries in the form of a novel BGC (top) or chemical compound (bottom) were embedded using their respective pre-trained foundation models. These embeddings were then projected by our trained BGC and chemical encoders into a common co-embedding space, where the nearest embeddings of the opposite modality (BGCs for chemical queries and chemicals for BGC queries) were retrieved using k-Nearest Neighbor search (k-NN), and ranked based on proximity.

We use BiGCARP^6^ (https://github.com/microsoft/bigcarp) to generate the initial 256-dimensional embeddings of BGCs and use MoLFormer^14^ (https://huggingface.co/ibm/MoLFormer-XL-both-10pct) to get the initial 768-dimensional embeddings of compounds. Both foundation models are masked language models trained on unlabeled data using self-supervised learning. BiGCARP represents BGCs as chains of functional protein domains and uses a convolutional masked language model to learn meaningful representations of BGCs and their constituent Pfam domains. MoLFormer is a transformer encoder based chemical language model trained on over one billion SMILES strings. It leverages a linear attention mechanism and rotary positional embeddings for efficient training, and is able to accurately predict a diverse range of chemical properties. Other BGC or chemical foundation models can be employed in our model to generate the initial embeddings of BGCs and compounds as well if they can help further improve model performance.

The initial embeddings of BGCs and compounds are mapped to *d*-dimensional embeddings in the co-embedding space via modality-specific encoders—one for BGCs and one for compounds. The two encoders share the same model architecture as shown in Fig. 1B but they do not share model weights. Within the two encoders, the initial embeddings generated by foundation models are linearly transformed first before they are passed through a two-layer transformer encoder. The linear transformation layer is defined as below, where $e_f$ is an initial embedding generated by a foundation model with dimension $d_f$, $W_1\in \mathcal {R}^{d_f\times d}$, $b_1$ is a *d*-dimensional vector. Both $W_1$ and $b_1$ are learnable parameters.

1 $$\begin{aligned} linear(e_f) = e_f \cdot W_1 + b. \end{aligned}$$

We use the standard transformer encoder^26^ in our model. The output vector of the transformer encoder is averaged along the sequence dimension to remove the sequence dimension and is then concatenated with the mean vector of the initial embedding sequence. The concatenation of the two vectors is then passed through a batch normalization layer and a two-layer multilayer perceptrons (MLP)^27^ to get the final *d*-dimensional embeddings in the co-embedding space. The MLP layer is defined as below, where *e* is the input vector to the MLP layer with dimension $d+d_f$, $W_2\in \mathcal {R}^{(d+d_f)\times d_h}$, $b_2$ is a $d_h$-dimensional vector, $W_3\in \mathcal {R}^{d_h\times d}$, $b_3$ is a *d*-dimensional vector. $W_2$, $b_2$, $W_3$ and $b_3$ are learnable parameters.

2 $$\begin{aligned} MLP(e) = Dropout(ReLU(e\cdot W_2+b_2))\cdot W_3 + b_3. \end{aligned}$$

*Model hyper-parameters*. The number of layers in transformer encoder and MLP is set to 2. The dimension of the co-embedding space *d* is set to 64. The hidden dimension of the feed-forward neural network in the transformer encoder and the MLP layer is set to 512, and the number of heads is set to 8. The length of the input sequences to the transformer encoder is set to 128 given that the sequence length of 92.6% of BGCs and 81.6% of compounds is no more than 128. If the sequence length of a BGC or a compound is larger than 128, we use the first length-128 sub-sequence only. We tested longer sequence length like 256 and it does not yield better performance than using 128.

After the embeddings of BGCs and compounds in the co-embedding space are generated, we can then use nearest neighbor search for efficient bi-directional retrieval of BGCs and compounds as illustrated in Fig. 2. The similarity between a BGC *b* and a compound *m* in the co-embedding space is measured using cosine similarity defined in Eq. (3), where $e^{b}$ is the embedding of BGC *b*, $e^m$ is the embedding of compound *m*, and *d* is the dimension of the co-embedding space. The higher the similarity between a BGC and a compound is, the more likely the compound is a natural product of the BGC.

3 $$\begin{aligned} \cos (b, m) = \frac{e^b \cdot e^m}{\Vert e^b\Vert \Vert e^m\Vert } = \frac{\sum _{i=1}^d e^b_i e^m_i}{\sqrt{\sum _{i=1}^d ({e^b_i})^2} \sqrt{\sum _{i=1}^d ({e^m_i})^2}}. \end{aligned}$$

#### Model training

During training, we freeze the initial embeddings generated by foundation models to preserve their learned representations, and only update the parameters of the two encoders. This strategy allows us to leverage the rich feature representations learned by the two foundation models while reducing the risk of over-fitting.

To align the embeddings of BGCs and compounds in the co-embedding space, we employ metric learning with N-pair loss^28^ to maximize the similarity between positive BGC-compound pairs and minimize the similarity of negative pairs. In each batch, *N* BGC-compound positive pairs $\mathcal {T}=\{(b_k, m_k)\}_{k=1}^N$ are randomly sampled from training data. Given a positive pair (*b*, *m*), N-pair loss with BGC *b* as the anchor is defined in Eq. (4) and N-pair loss with compound *m* as the anchor is defined in Eq. (5), where $\mathcal {M}_{b, \mathcal {T}}^{-}$ is the set of negative compounds of BGC *b* from $\mathcal {T}$, $\mathcal {B}_{m,\mathcal {T}}^-$ is the set of negative BGCs of compound *m* from $\mathcal {T}$, and $\cos (b, m)$ is the cosine similarity defined in Eq. (3). Note that negative BGCs of *m* and negative compounds of *b* are taken from other pairs in the same batch. A factor of 5 is multiplied to $\cos (b, m^{-}) - \cos (b,m)$ because it penalizes the difference more which leads to better performance than not multiplying in our experiments. The main reason for the performance improvement with a factor of 5 is that the value range of cosine similarity is [-1, 1]. Multiplying a factor of 5 extends the value range to [-5, 5], which enables the model to learn more effectively from the difference between $\cos (b, m^{-})$ and $\cos (b,m)$. The N-pair loss over batch $\mathcal {T}$ is the mean of $L_{N-pair}^b(b, m, \mathcal {M}_{b,\mathcal {T}}^-)$ and $L_{N-pair}^m(m, b, \mathcal {B}_{m,\mathcal {T}}^-)$ over all positive pairs in $\mathcal {T}$ as given in Eq. (6).

4 $$\begin{aligned} L_{N-pair}^b(b, m, \mathcal {M}_{b, \mathcal {T}}^-) = \log (1 + \sum _{m^{-} \in \mathcal {M}_{b, \mathcal {T}}^{-}} \exp (5(\cos (b, m^{-}) - \cos (b,m)))), \end{aligned}$$

5 $$\begin{aligned} L_{N-pair}^m(m, b, \mathcal {B}_{m, \mathcal {T}}^-) = \log (1 + \sum _{b^{-} \in \mathcal {B}_{m, \mathcal {T}}^{-}} \exp (5(\cos (b^{-}, m) - \cos (b, m)))), \end{aligned}$$

6 $$\begin{aligned} L_{N-pair}(\mathcal {T}) = \frac{1}{2N}\sum _{k=1}^{N}(L_{N-pair}^b(b_k, m_k, \mathcal {M}_{b_k,\mathcal {T}}^-) + L_{N-pair}^m(m_k, b_k, \mathcal {B}_{m_k,\mathcal {T}}^-)). \end{aligned}$$

The model is trained using the Adam optimizer to minimize $L_{N-pair}(\mathcal {T})$ in each batch. To achieve that, the optimizer needs to push negative pairs apart by reducing $cos(b, m^{-})$ and $cos(b^{-}, m)$ and draw positive pairs close by increasing *cos*(*b*, *m*). Our model is trained using one cycle of cosine annealing schedule with an initial learning rate of 0.003 and a minimum learning rate of 0.0001. The number of epochs in one cycle is set to 100. The dropout rate is set to 0.1 for dropout layers in transformer encoders and MLP. A dropout layer is a regularization technique used in deep learning to prevent overfitting and improve model generalization.

Besides using N-pair loss, we also tested two other alternative loss functions for comparison: Binary Cross Entropy (BCE) loss and triplet loss. To calculate BCE loss over a batch $\mathcal {T}=\{(b_k, m_k)\}_{k=1}^N$, we randomly sample a negative batch $\mathcal {T}^-=\{(b_k^-, m_k^-)\}_{k=1}^N$ where $(b_k^-, m_k^-) \notin \mathcal {D}$. For each pair (*b*, *m*) in $\mathcal {T} \cup \mathcal {T}^-$, we directly estimate the probability whether (*b*, *m*) is positive or not as given in Eq. (7) below where $\sigma$ is the sigmoid function. Here we multiply a factor of 5 to $\cos (b, m)$ to extend the value range of $\hat{y_x}$ from [0.2689, 0.7311] to [0.0067, 0.9933].

7 $$\begin{aligned} \hat{y}_{(b,m)} = Prob((b,m)\text { is a positive pair}) = \sigma (5\cos (b, m)). \end{aligned}$$

BCE loss over $\mathcal {T} \cup \mathcal {T}^-$ is defined in Eq. (8).

8 $$\begin{aligned} L_{BCE} (\mathcal {T} \cup \mathcal {T}^-) = -\frac{1}{2N}(\sum _{(b,m)\in \mathcal {T}}\log (\hat{y}_{(b,m)}) + \sum _{(b^-, m^-)\in \mathcal {T}^-}\log (1-\hat{y}_{(b^-, m^-)})). \end{aligned}$$

Triplet loss is similar to N-pair loss, but it is calculated on one negative pair only instead of N-1 negative pairs. Given a positive pair (*b*, *m*), triplet loss with BGC *b* as the anchor is defined in Eq. (9) and triplet loss with compound *m* as the anchor is defined in Eq. (10), where $b^-$ and $m^-$ are randomly sampled such that $(b^-, m)\notin \mathcal {D}$ and $(b, m^-)\notin \mathcal {D}$. The triplet loss over batch $\mathcal {T}$ is the mean of $L_{triplet}^b(b, m, m^-)$ and $L_{triplet}^m(m, b, b^-)$ over all positive pairs in $\mathcal {T}$ as given in Eq. (11).

9 $$\begin{aligned} L_{triplet}^b(b, m, m^-) = \log (1 + \exp (5(\cos (b, m^{-}) - \cos (b,m)))), \end{aligned}$$

10 $$\begin{aligned} L_{triplet}^m(m, b, b^-) = \log (1 + \exp (5(\cos (b^{-}, m) - \cos (b, m)))), \end{aligned}$$

11 $$\begin{aligned} L_{triplet}(\mathcal {T}) = \frac{1}{2N}\sum _{k=1}^{N}(L_{triplet}^b(b_k, m_k, m_k^-) + L_{triplet}^m(m_k, b_k, b_k^-)). \end{aligned}$$

Our model and all baseline models below are implemented using Python 3.11, PyTorch 2.5.1 and pytorch-cuda 12.1.

### Baseline: K-nearest neighbor search without alignment

We compare our BCCoE model with a more conventional approach of k-nearest neighbor search in the original embedding space directly without alignment. The assumption behind this baseline approach is that if two BGCs are very similar to each other, then they are likely to produce the same or similar natural products; and if two compounds are very similar to each other, then they are likely to be produced by the same or similar BGCs. We implemented two versions of this baseline, KNN and KNN-2hop, and both of them use cosine similarity over the original embeddings generated by BGC and chemical foundation models. The difference between the two is that KNN uses either similarities between BGCs or similarities between compounds depending on the query while KNN-2hop uses both similarities in order to retrieve novel BGCs or compounds not in the training data.

**Fig. 3:**
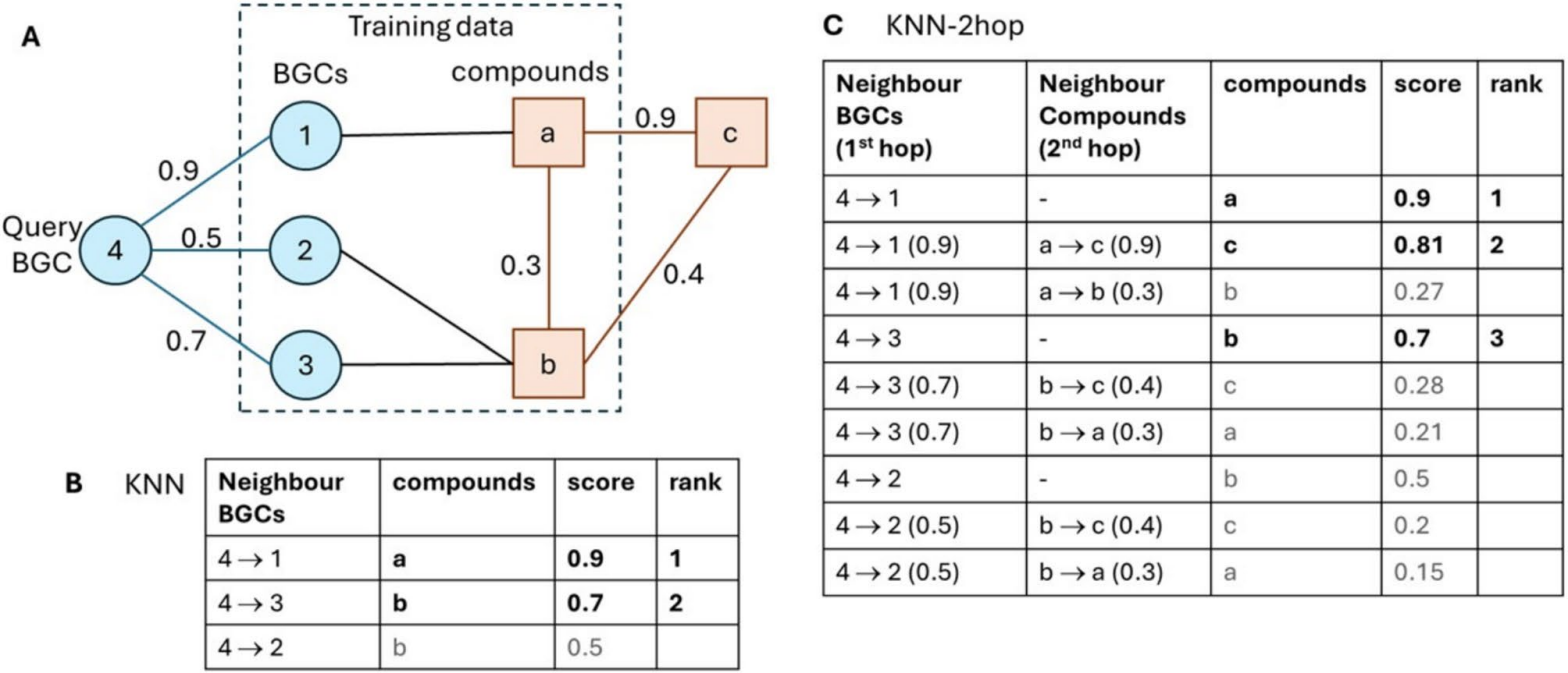
KNN and KNN-2hop. (**A**) An example training data. Similarities between the query BGC (BGC 4) and BGCs in the training data are calculated using the initial embeddings generated by a BGC foundation model. Similarities between compounds are calculated using the initial embeddings generated by a chemical foundation model. (**B**) compounds retrieved and ranked by KNN for the given query BGC. (**C**) compounds retrieved and ranked by KNN-2hop for the query BGC.

Figure 3A shows an example training data containing three positive BGC-compound pairs: (1, a), (2, b) and (3, b), where 1, 2, 3 are BGCs and a, b, c are compounds. Given a query BGC 4, the similarity between BGC 4 and the three BGCs in the training data are calculated over the initial BGC embeddings. Among the three BGCs in the training data, BGC 1 has the highest similarity of 0.9 with the query BGC, so its compound, compound a, is ranked first. KNN is unable to retrieve and rank compound c because compound c is not produced by any BGC in the training data. KNN-2hop overcomes this limitation by considering the similarities between compounds in addition to similarities between BGCs. In KNN-2hop, the route from the query BGC to a compound can be either through a neighbor BGC as in KNN or through a neighbor BGC (the first hop) plus a neighbor compound (the second hop). In the latter case, the final score is the product of the two similarities over the two hops. For both KNN and KNN-2hop, if there are multiple routes from a query BGC and a compound, the one with the highest score is used, and other routes are ignored. In the example in Fig. 3, KNN-2hop is able to retrieve and rank compound c for the query BGC. Among the three routes from the query BGC to compound c, the one containing BGC 1 and compound a has the highest final score of 0.81, so this route is used and the other two routes are ignored. Figure 3 shows how compounds are retrieved and ranked for a given query BGC using KNN and KNN-2hop. The opposite direction of retrieving BGCs for a given query compound is done in a similar way.

### Baseline: predicting based on product classes

Both BCCoE and the two KNN methods use BGC sequence, SMILES and foundation models to prioritize compounds/BGCs for given queries. We also include a much simpler baseline called SameClass which prioritizes compounds/BGCs based on the product classes of queries. More specifically, given a query and a candidate pool, SameClass ranks compounds/BGCs from the same class as the query in the front followed by compounds/BGCs in other classes. The order of the compounds/BGCs within same classes are random.

### Datasets

We used MIBiG version 3.1 and 4.0 (https://mibig.secondarymetabolites.org/download) in our study. Only active BGCs and their natural products are included. BGCs are represented by sequences of Pfam domains, which are generated from protein FASTA sequences of BGCs using HMMER version 3.4 and Pfam 31.0 model. Compounds are represented by their canonical SMILES. Non-canonical SMILES are converted to canonical SMILES using MolToSmiles() function from rdkit.Chem with canonical=True. BGCs without Pfam domains or associated SMILES are excluded. The first two rows in Table 1 shows the number of active BGCs with Pfam sequences, the number of compounds with SMILES, BGC-compound pairs, number of BGCs in pairs and number of compounds in pairs in the two versions. The last two rows in Table 1 shows the number of BGCs, compounds and BGC-compound pairs in version 3.1 only or in version 4.0 only.

**Table 1:** Statistics of MIBiG version 3.1 and version 4.0.

Dataset	#BGCs	#compounds	#pairs	#BGCs	#compounds
				in pairs	in pairs
version 3.1	2494	2688	3110	1906	2682
version 4.0	2625	3000	3422	1987	2992
only in 3.1	88	120	161	53	120
only in 4.0	219	432	473	134	430

### Model evaluation

For a given query BGC *b* and a list of candidate compounds, BCCoE ranks the compounds by their similarity to *b* in the co-embedding space. The higher the similarity, the more likely the compounds are produced by BGC *b*. Similarly, given a query compound *m* and a list of candidate BGCs, BCCoE ranks the BGCs in descending order of their similarity to *m*. We evaluate our model using number of hits, recall and lift at top-K, which are commonly used to evaluate the performance of information retrieval algorithms. We split data in $\mathcal {D}$ into training data and testing data. Training data is used to learn the embeddings of BGCs and compounds in the co-embedding space, and testing data is used as the ground-truth to calculate number of hits, recall and lift at top-K. If a BGC or a compound in the top-K actually occurs in the ground-truth set, then it is called a *hit*. Recall at top-K is defined as the number of hits among top-K predictions over the number of ground-truth pairs as given in Eq. (12).

12 $$\begin{aligned} \text {Recall}@\text {K} = \frac{{\#}\text {hits}@\text {K}}{{\#}\text {ground-truths}}. \end{aligned}$$

Lift is calculated by comparing the number of hits of a model with the number of hits of a random guessing method as given in Eq. (13). For the random guessing method, we use its average performance over 100 runs to ensure reliable measurements.

13 $$\begin{aligned} \text {Lift}@\text {K} = \frac{{\#}\text {hits}@\text {K}}{{\#}\text {hits}@\text {K of random guessing}}. \end{aligned}$$

### Three experimental settings for bidirectional retrieval

Under *10-fold cross-validation*, MIBiG 4.0 dataset is split randomly either based on BGCs or compounds into 10 folds. If the task is to retrieve compounds for a given BGC, then data are split based on BGCs randomly so that a BGC and all its positive pairs are in one and only one fold (this experiment is denoted as “exp 1”); if the task is to retrieve BGCs for a given compound, then data are split based on compounds randomly so that a compound and all its positive pairs are in one and only one fold (denoted as “exp 2”). Then each fold is used as testing data, and the remaining nine folds are used as training data to learn the model parameters.

*Holding out one BGC product class* aims to test the ability of our model in extrapolating to novel product classes. MIBiG 4.0 dataset is used for this experiment. Table 2 shows the list of product classes in MIBiG 4.0, number of BGCs, number and percentage of positive pairs involving the BGCs, number of compounds, number and percentage of positive pairs involving the compounds in each product class. As BGCs or compounds can belong to more than one class, the sum of the percentages is more than 100%. We use each of these classes as a target class. If the task is to retrieve compounds for a given BGC, we split based on BGCs and use the BGCs of the target class and their positive pairs as the testing data (denoted as “exp 3”). If the task is to retrieve BGCs for a given compound, we split based on compounds and use compounds of the target class and their positive pairs as the testing data (denoted as “exp 4”). The remaining data are used as training data. For both splits, the training data do not contain any testing BGC/compound from the target class. The model only has access to the training data, so the target class is novel to the model. This data splitting strategy represents a very challenging situation for machine learning because the training data and the testing data have very distinct distributions.

**Table 2:** Statistics of BGC product classes.

Classes	#BGCs	BGC pairs		#compounds	Compound pairs	
		num	%		Num	%
PKS	932	1673	48.9%	1483	1697	49.6%
NRPS	737	1377	40.2%	1242	1402	41.0%
Ribosomal	142	184	5.4%	173	184	5.4%
Saccharide	141	202	5.9%	152	227	6.6%
Terpene	152	292	8.5%	263	307	9.0%
Other	335	592	17.3%	520	611	17.9%

*Using MIBiG 3.1 to predict new pairs in MIBiG 4.0* represents a realistic situation where we use known positive pairs to predict novel new pairs. There are 473 new BGC-compound pairs in MIBiG 4.0 compared with MIBiG 3.1 as shown in Table 1. We further divide these new pairs based on whether their BGCs and compounds exist in MIBiG 3.1 or are new in MIBiG 4.0 as shown in Table 3. The total number of unique BGCs in the new pairs is 196 and the total number of unique compounds in the new pairs is 457. If the task is to retrieve compounds for given BGCs, we use the 196 BGCs in the new pairs as query BGCs, and use either the compounds in MIBiG 3.1 (denoted as “exp 5”) or the compounds in MIBiG 4.0 (denoted as “exp 7”) as the candidate pool to retrieve compounds from. If the task is to retrieve BGCs for given compounds, we use the 457 compounds in the new pairs as query compounds, and use either the BGCs in MIBiG 3.1 (denoted as “exp 6”) or the BGCs in MIBiG 4.0 (denoted as “exp 8”) as the candidate pool to retrieve BGCs from. Note that when the BGCs or compounds from MIBiG 4.0 are used as candidates for retrieval, the BCCoE model is still trained using MIBiG 3.1 dataset only. The candidate BGCs and compounds from MIBiG 4.0 are for evaluation only.

**Table 3:** #new pairs with existing or new BGCs/compounds.

	Existing BGCs	New BGCs	Total
Existing compounds	25	4	29
New compounds	404	40	444
Total	429	44	473

For all the three settings above, there is strictly no overlapping of BGC-compound pairs between testing data and training data, and testing data contain novel BGCs/compounds not in the training data. The percentage of testing pairs (ground-truths) involving novel BGCs and compounds under each of the eight experiments are provided in the last two columns of Table 4. For both 10-fold cross-validation and holding out one product class settings, if the task is to rank compounds for given query BGCs, training and testing data are split based on BGCs, so all the BGCs in testing data are novel in “exp 1” and “exp 3”; if the task is to rank BGCs for given query compounds, training and testing data are split based on compounds, so all the compounds in testing data are novel in “exp 2” and “exp 4”.

**Table 4:** Coverage and percentage of novel BGCs and compounds in the 8 experiments.

Exp	Setting	Candidate	#test	% of test pairs		
		Pool	Pairs	Candidate pool coverage	Novel BGCs	Novel compounds
1	10-fold CV, BGC2Chem	Compounds in MIBiG 4.0	3422	100%	100%	81.3%
2	10-fold CV, Chem2BGC	BGCs in MIBiG 4.0	3422	100%	43.0%	100%
3	BGC classes, BGC2Chem	Compounds in MIBiG 4.0	4320	100%	100%	97.7%
4	BGC classes, Chem2BGC	BGCs in MIBiG 4.0	4428	100%	99.3%	100%
5	3.1$\rightarrow$4.0, BGC2Chem	Compounds in MIBiG 3.1	473	6.1%	9.3%	93.9%
6	3.1$\rightarrow$4.0, Chem2BGC	BGCs in MIBiG 3.1	473	90.7%	9.3%	93.9%
7	3.1$\rightarrow$4.0, BGC2Chem	Compounds in MIBiG 4.0	473	100%	9.3%	93.9%
8	3.1$\rightarrow$4.0, Chem2BGC	BGCs in MIBiG 4.0	473	100%	9.3%	93.9%

The percentage of testing (ground-truth) pairs that are covered by the candidate pool is shown in the 3rd last column of Table 4. For both 10-fold cross-validation and holding out one product class settings, the candidate BGCs and compounds are from MIBiG 4.0. Given that the testing data are subsets of MIBiG 4.0 dataset, so all the compounds/BGCs in the testing pairs are covered by the candidate pool under these two settings. When using MIBiG 3.1 data to predict new pairs in MIBiG 4.0, if we assume that MIBiG 3.1 data is all we have and strictly use only data in MIBiG 3.1 to form candidate pool (in “exp 5” and “exp 6”), then some testing pairs cannot be covered by the candidate pool. For example, in “exp 5”, only 6.1% of testing pairs can be covered by compounds in MIBiG 3.1, and the recall of retrieval algorithms is thus upper-bounded by 6.1%. If we expand the candidate pool using the BGCs and compounds from MIBiG 4.0 (in “exp 7” and “exp 8”), then all the testing pairs can be covered by the candidate pool.

## Results

In this section, we report the performance of our co-embedding framework across three settings designed to test its generalizability: (i) 10-fold cross-validation, (ii) holding out one product class where a specific compound class were excluded from training, and (iii) temporal generalization, where models trained on MIBiG version 3.1 were used to predict novel links in the subsequently released MIBiG version 4.0. These evaluation settings reflect different real-world deployment scenarios, including exploiting known compound classes, extrapolation to new classes, and forward prediction of unseen genomic-chemical links.

We compare our model with Random, SameClass, KNN and KNN-2hop methods described above. Random represents the situation when no knowledge of BGCs and compounds are used for bidirectional retrieval. SameClass represents the situation when some knowledge of BGCs and compounds such as their classes is used. The two KNN methods represent the situation that the same amount of information of BGCs and compounds are used as our proposed BCCoE model, but there is no alignment in the co-embedding space. Together, the four baseline methods span a spectrum of bidirectional retrieval approaches. The variation in their performance highlights the extent of improvement gained by incorporating additional information and by enforcing alignment within the co-embedding space.

**Table 5:** #hits, Recall and Lift of different models for bidirectional retrieval.

Exp	Setting	top-K	Random		SameClass			KNN			KNN-2hop			BCCoE		
			#hits	Recall	#hits	Recall	Lift	#hits	Recall	Lift	#hits	Recall	Lift	#hits	Recall	Lift
1	10-fold CV	10	11.4	0.3%	49.7	1.5%	4.4	443	12.9%	39.0	748	21.9%	65.8	1125	32.9%	98.9
	BGC2Chem	100	114.7	3.4%	495.0	14.5%	4.3	521	15.2%	4.5	1422	41.6%	12.4	2243	65.5%	19.6
2	10-fold CV	10	12.6	0.4%	58.7	1.7%	4.7	1836	53.7%	145.7	2075	60.6%	164.7	2235	65.3%	177.4
	Chem2BGC	100	128.5	3.8%	577.9	16.9%	4.5	1900	55.5%	14.8	2431	71.0%	18.9	2864	83.7%	22.3
3	BGC classes	10	14.9	0.3%	53.6	1.2%	3.6	60	1.4%	4.0	145	3.4%	9.7	253	5.9%	17.0
	BGC2Chem	100	145.6	3.4%	545.3	12.6%	3.7	72	1.7%	0.5	478	11.1%	3.3	1063	24.6%	7.3
4	BGC classes	10	16.5	0.4%	67.2	1.5%	4.1	25	0.6%	1.5	176	4.0%	10.7	333	7.5%	20.2
	Chem2BGC	100	166.1	3.8%	662.1	14.9%	4.0	28	0.6%	0.2	502	11.3%	3.0	1333	30.1%	8.0
5	3.1$\rightarrow$4.0	10	0.1	0.0%	0.3	0.1%	4.7	14	3.0%	200.0	16	3.4%	228.6	23	4.9%	328.6
	BGC2Chem3.1	100	1.0	0.2%	4.0	0.8%	3.9	21	4.4%	20.4	24	5.1%	23.3	25	5.3%	24.3
6	3.1$\rightarrow$4.0	10	1.9	0.4%	7.2	1.5%	3.8	127	26.8%	67.9	168	35.5%	89.8	188	39.7%	100.5
	Chem2BGC3.1	100	17.6	3.7%	76.8	16.2%	4.4	146	30.9%	8.3	223	47.1%	12.7	313	66.2%	17.8
7	3.1$\rightarrow$4.0	10	1.7	0.4%	6.4	1.3%	3.8	14	3.0%	8.2	126	26.6%	74.1	180	38.1%	105.9
	BGC2Chem4.0	100	15.7	3.3%	61.3	12.9%	3.9	21	4.4%	1.3	230	48.6%	14.6	329	69.6%	20.9
8	3.1$\rightarrow$4.0	10	1.8	0.4%	8.2	1.7%	4.7	127	26.8%	72.2	168	35.5%	95.5	190	40.2%	108.0
	Chem2BGC4.0	100	17.8	3.8%	82.5	17.4%	4.6	146	30.9%	8.2	236	49.9%	13.3	335	70.8%	18.8

**Fig. 4:**
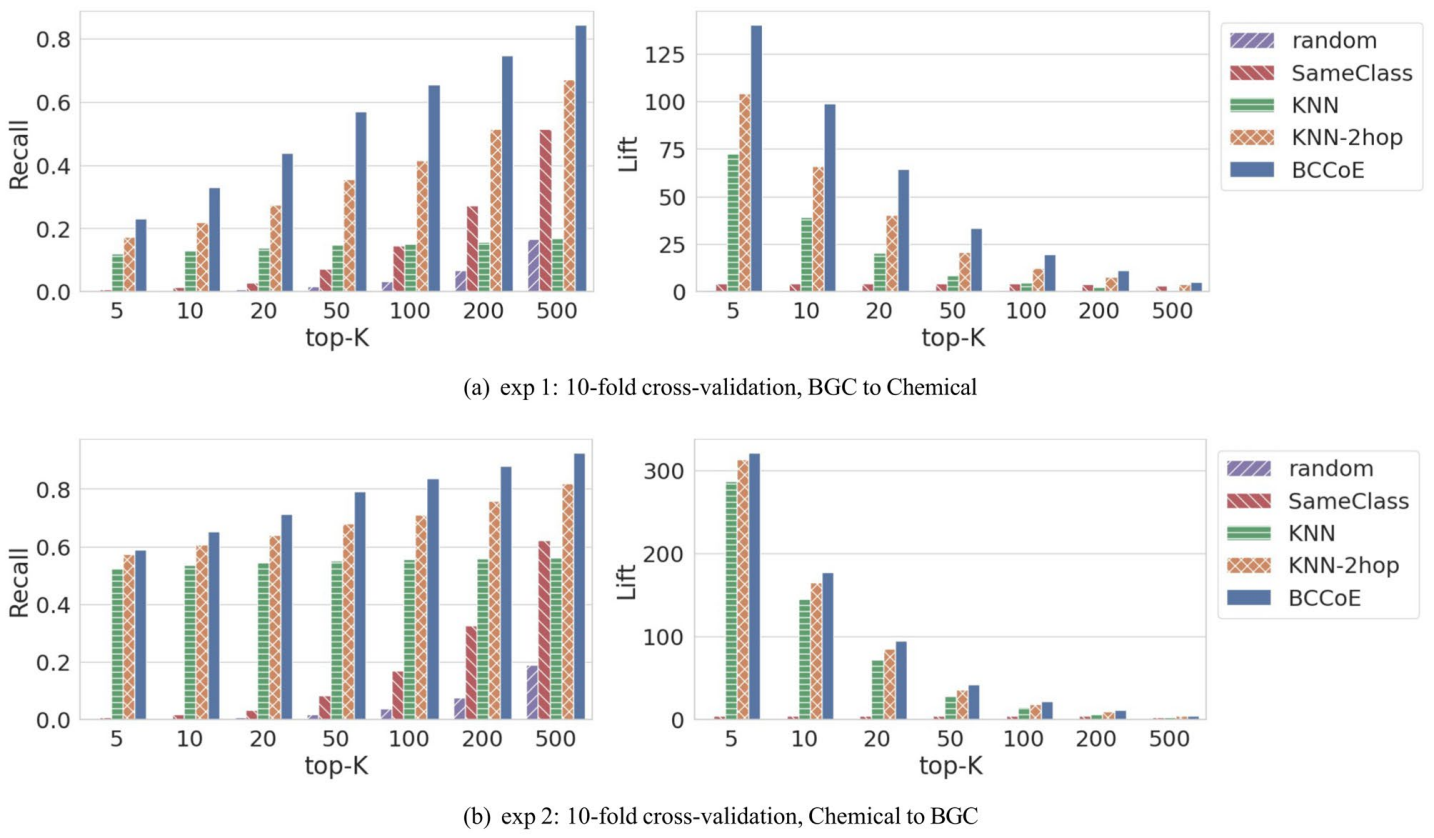
Model performance for bidirectional retrieval when using 10-fold cross-validation: Recall (left) and Lift (right).

**Fig. 5:**
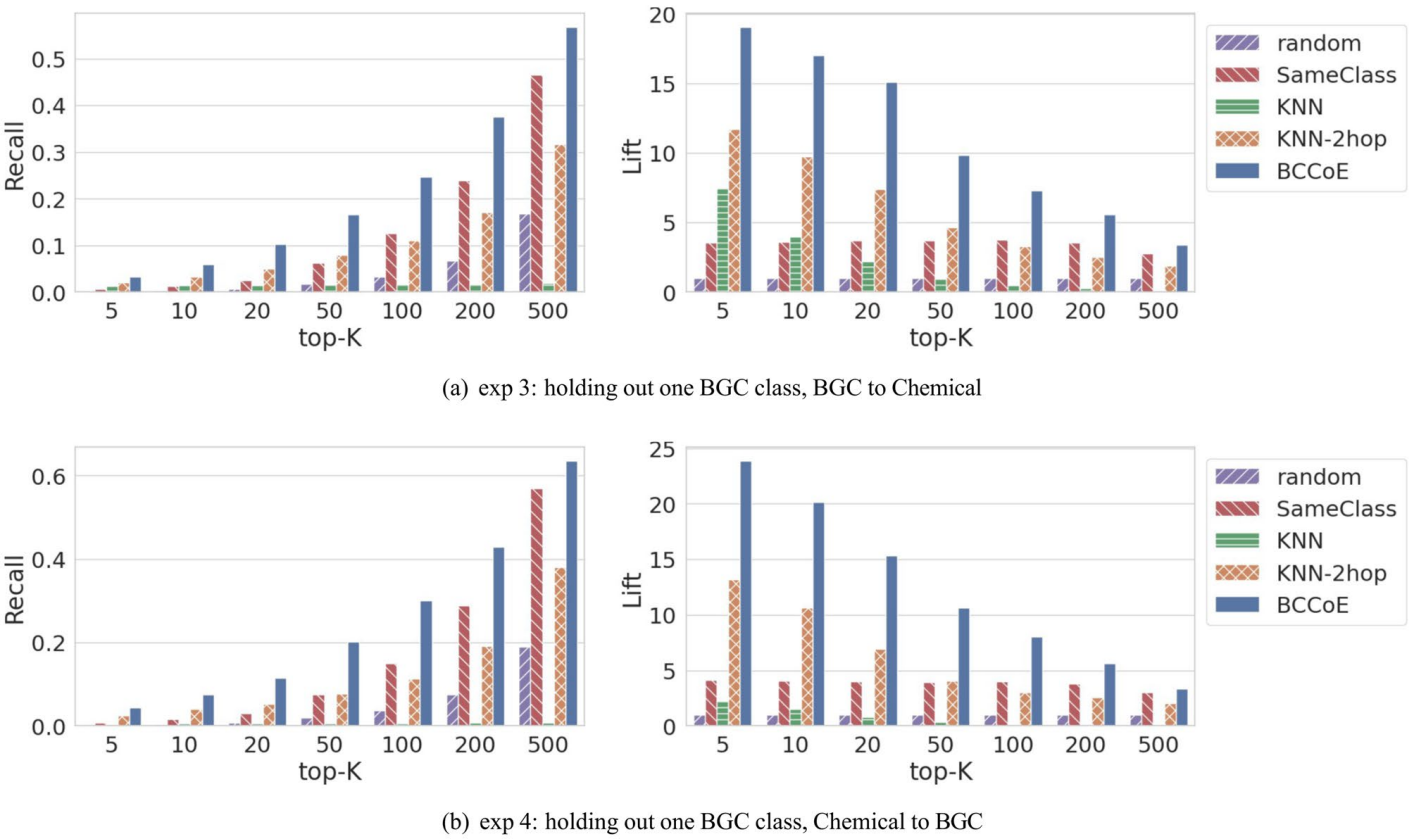
Model performance for bidirectional retrieval when holding out one product class: Recall (left) and Lift (right).

**Fig. 6:**
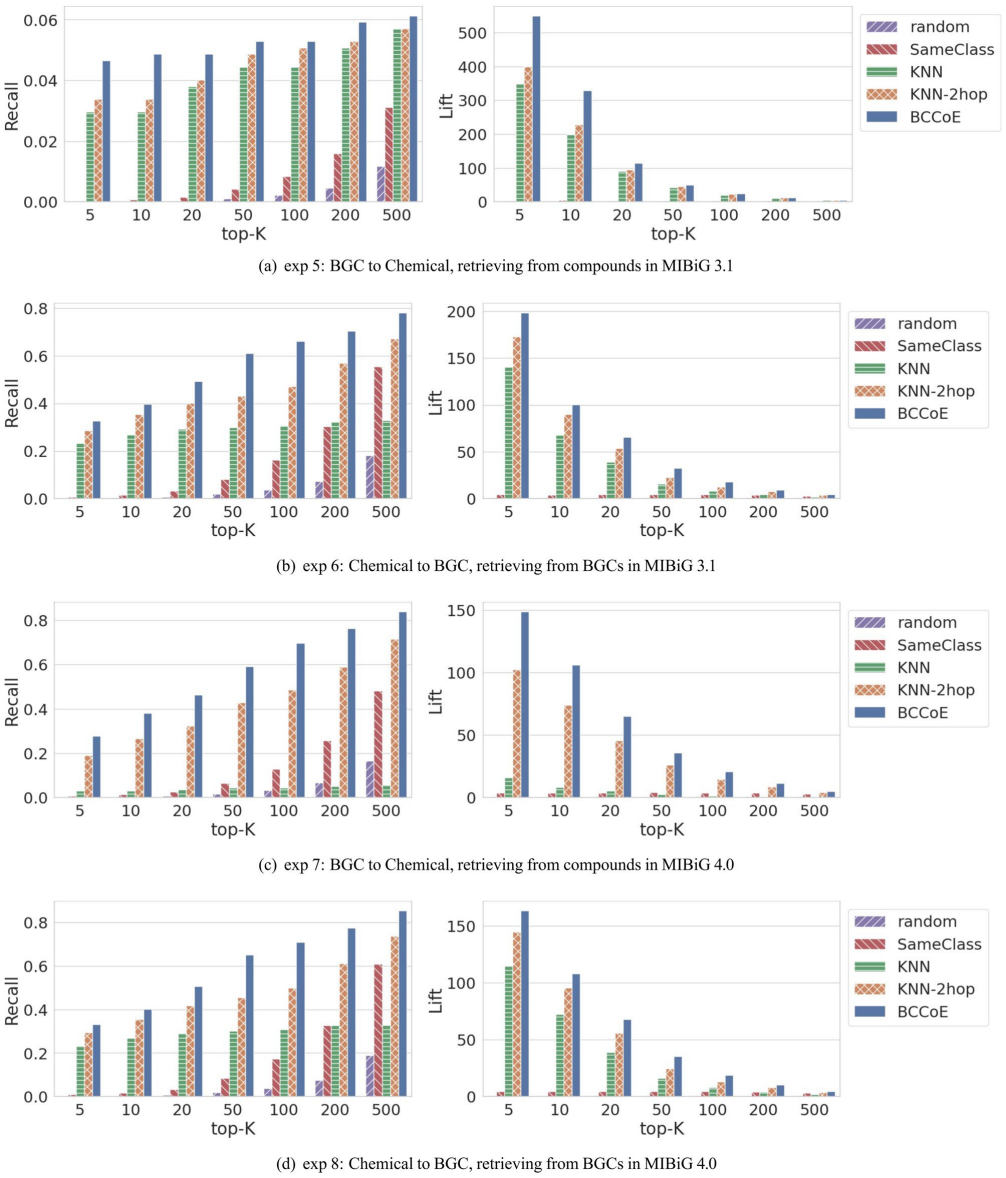
Model performance when using MIBiG 3.1 to predict new pairs in MIBiG 4.0: Recall (left) and Lift (right).

**Fig. 7:**
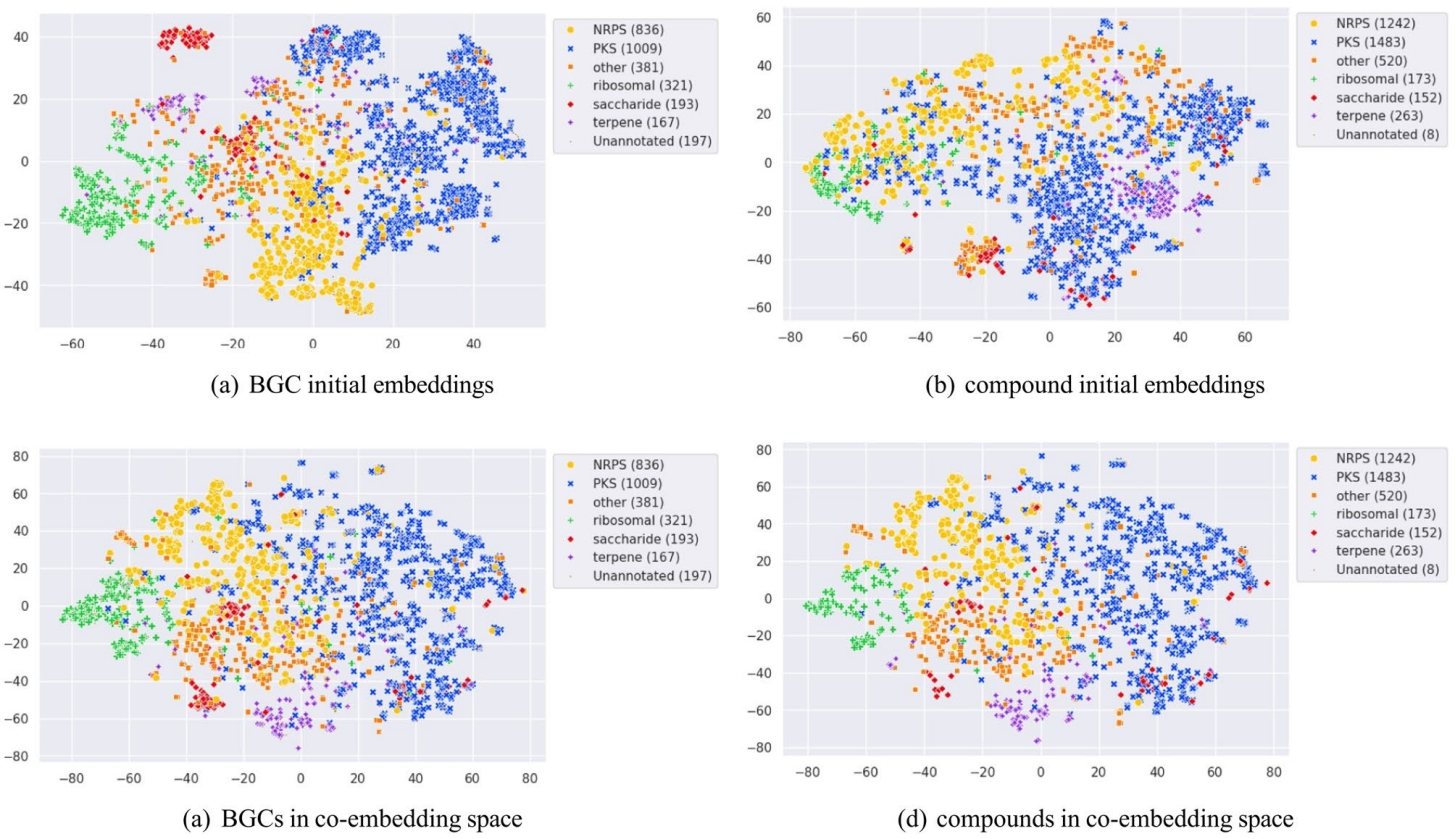
Visualization of BGCs and compounds: initial embeddings (top) and their embeddings in the co-embedding space (bottom).

### Model performance under 10-fold cross-validation

The performance of the models under 10-fold cross-validation is shown in Fig. 4 and “exp 1” row and “exp 2” row in Table 5 where the performance of the models is aggregated over the 10 folds. Our model with N-pair loss shows a very high enrichment compared with random guessing especially when K is small. The SameClass method is less than 5 times better than random guessing because it can only differentiate different classes, but cannot differentiate different BGCs/compounds within the same class. The BCCoE model shows much better performance than the two KNN methods when the task is to retrieve compounds for given BGCs. At top-10, KNN retrieves 12.9% of ground-truths, KNN-2hop retrieves 21.9% of ground-truths, which is a 68.8% improvement over KNN, and BCCoE is able to pick up 32.9% of ground-truths, which is a further improvement of 50.4% over KNN-2hop. When the task is to retrieve BGCs for given compounds, both BCCoE and KNN-2hop perform very well. They can pick up 65.3% and 60.6% of the ground-truths respectively even at just top-10. In comparison, random guessing can only pick up 0.4% of the ground-truths at top-10.

### Model performance when holding-out one product class

The performance of the models under this setting is shown in Fig. 5 and “exp 3” row and “exp 4” row in Table 5 where the performance of the models is aggregated over all the product classes. Compared with using 10-fold cross-validation, the performance of all models drops significantly in these two experiments as expected. Nevertheless, our model using N-pair loss can still achieve a lift of 17.0 and 20.2 at top-10 over random guessing for the two retrieval tasks respectively, which is 74.5% and 89.2% better over KNN-2hop respectively. This results highlight the versatility of the representations in the co-embedding space in identifying meaningful associations between BGCs and chemicals, even in under-explored biological and chemical spaces.

### Model performance when using MIBiG 3.1 to predict new pairs in MIBiG 4.0

The performance of the models for predicting new pairs in MIBiG 4.0 is shown in Fig. 6 and the last 8 rows in Table 5. Among the 473 new pairs, only 29 of them involving existing compounds in MIBiG 3.1. In “exp 5” where compounds are retrieved from MIBiG 3.1, KNN-2hop can retrieve 16 out of the 29 new pairs at top-10 while BCCoE is able to pick up 23 at top-10, which is a 43.8% improvement. This number increases to 126 (26.6%) and 180 (38.1%) respectively for the two models when retrieving from compounds from MIBiG 4.0 (“exp 7”). Most of the new pairs involving existing BGCs. BCCoE is able to pick up 188 (39.7%) new pairs at top-10 when retrieving from existing BGCs from MIBiG 3.1 (“exp 6”).

Together, the above results demonstrate the robustness and utility of our co-embedding model in both well-studied and emerging discovery settings. The SameClass method can be 6 times better than random guess at best given there are 6 product classes, while our model and the two KNN algorithms can be hundreds of times better than random guess at smaller k because they not only distinguishes different classes but also distinguishes compounds/BGCs within the same class based on the representations learned from BGC sequences and SMILES. Across all three evaluation paradigms, BCCoE consistently outperformed the two KNN algorithms which do not align embeddings in a co-embedding space, particularly at low K values, where enrichment is critical for practical screening. These improvements suggest that aligning genomic and chemical representations in a shared latent space can substantially streamline target prioritization, enabling more focused experimental validation and accelerating the discovery of novel natural products.

### Visualizing alignment in the learned co-embedding space

To qualitatively evaluate the extent of cross-modal alignment achieved by our co-embedding framework, we visualize the latent representations of BGCs and compounds from MIBiG 4.0 using two-dimensional projections generated by t-distributed stochastic neighbor embedding (t-SNE)^29^. The results are shown in Fig. 7. Prior to alignment, the initial embeddings produced by BiGCARP and MoLFormer displayed distinct clustering patterns within each modality. Both BGC and compound embeddings exhibited some grouping according to BGC product classes, indicating that the foundational models capture meaningful biochemical signals from Pfam domain sequences and SMILES strings, respectively. However, the embeddings of BGCs and compounds are in unrelated embedding space, limiting their utility for direct comparison or retrieval.

Following training with our deep metric learning objective, the joint co-embedding space exhibited markedly cross-modal alignment. As shown in Fig. 7c and d, BGCs and their corresponding compounds now occupy overlapping regions of the space, consistent with the model’s objective to co-locate positive BGC-compound pairs. These aligned representations not only facilitate bidirectional retrieval—as quantitatively demonstrated in prior sections—but also exhibit structure consistent with chemical class, suggesting potential utility for clustering, classification, and exploratory analyses. This visualization reinforces the interpretability and coherence of the learned latent space, supporting its application to both supervised and unsupervised tasks in natural product discovery.

We have used 10 nearest neighbours to predict the classes of BGCs and compounds in both original embedding spaces and the co-embedding space. The prediction accuracy is 0.867 for BGCs and 0.590 for compounds in the two original embedding spaces. In the common co-embedding space, the accuracy is increased to 0.884 for BGCs and 0.608 for compounds, indicating the product class clustering are well preserved in the co-embedding space.

**Fig. 8:**
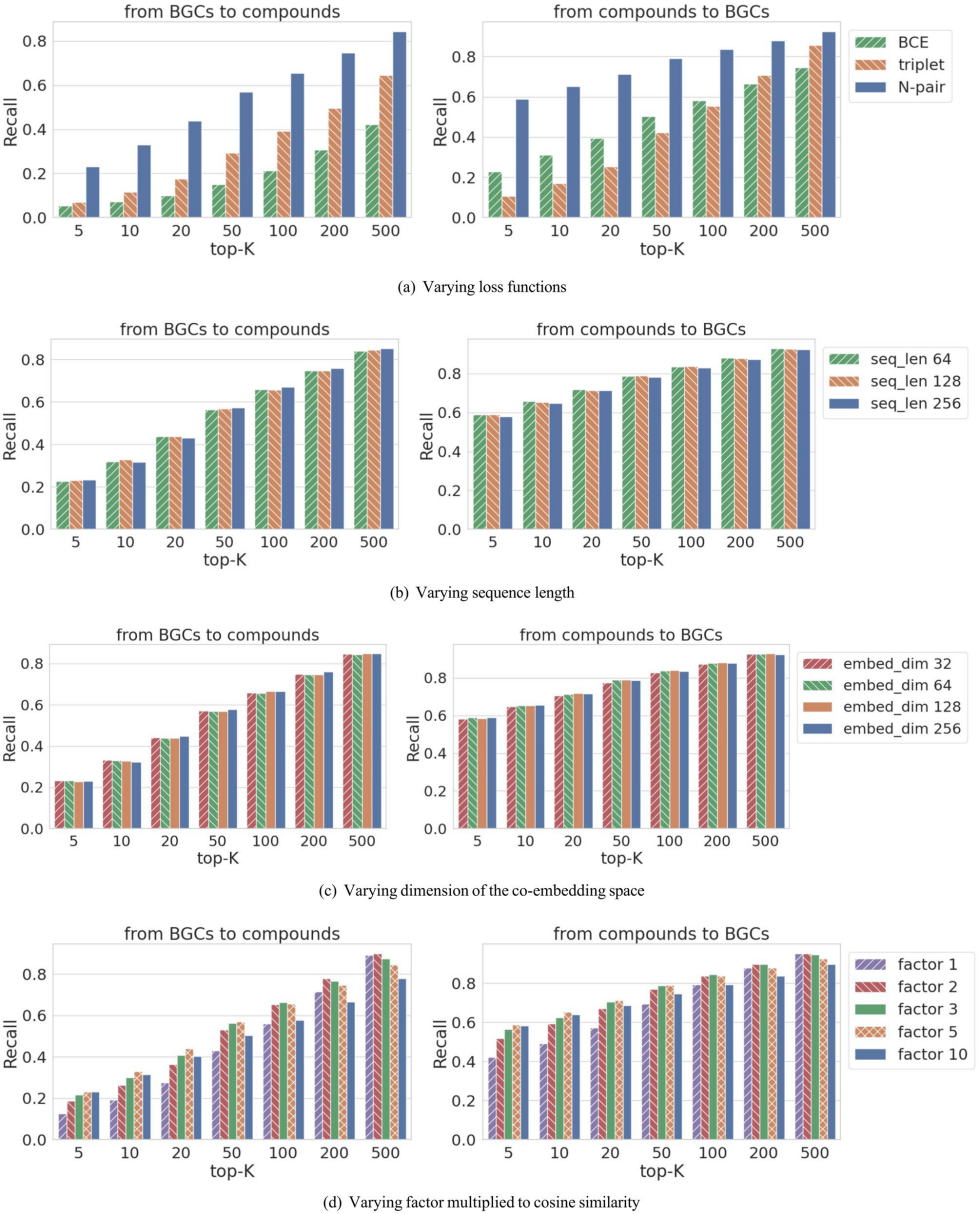
Recall of BCCoE for bidirectional retrieval when varying values of hyper-parameters: (**a**) loss function, (**b**) sequence length, (**c**) dimension of co-embedding space and (**d**) factor multiplied to cosine similarity.

**Fig. 9:**
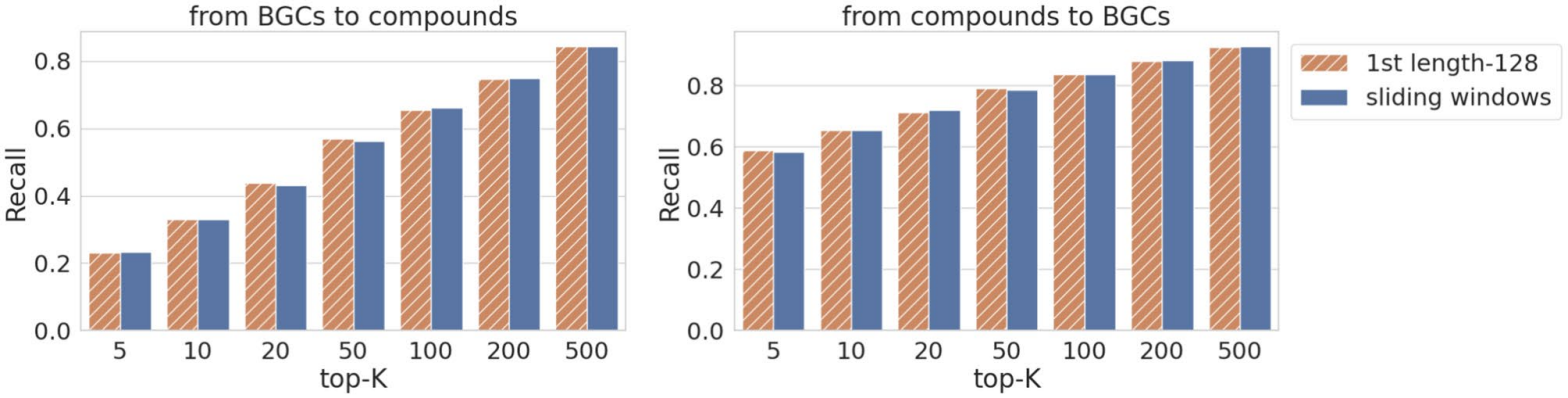
Recall of BCCoE for bidirectional retrieval with different sub-sequence sampling strategies.

**Fig. 10:**
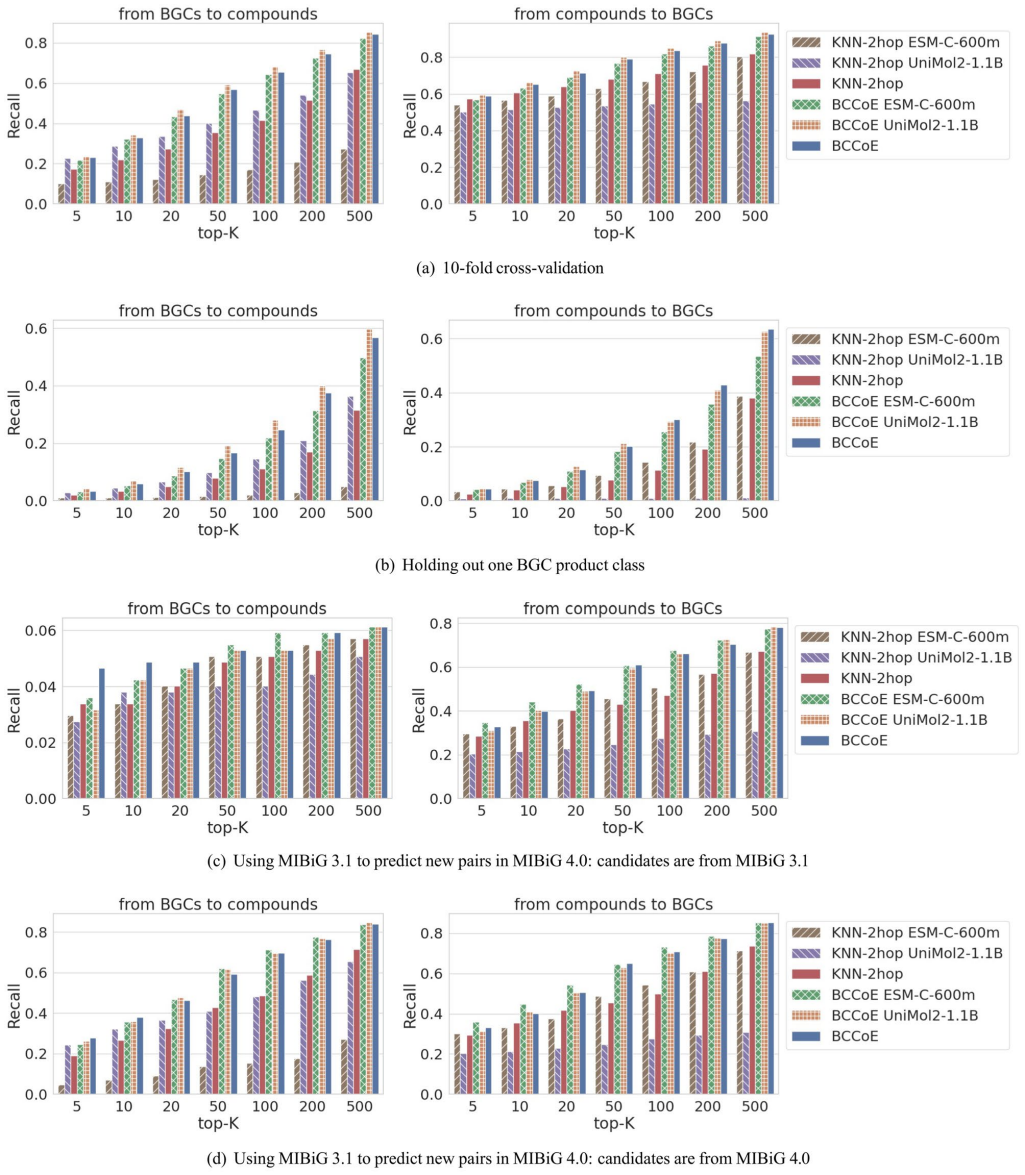
Recall of models for bidirectional retrieval when using different foundation models. Left: from BGCs to compounds; Right: from compounds to BGCs.

**Fig. 11:**
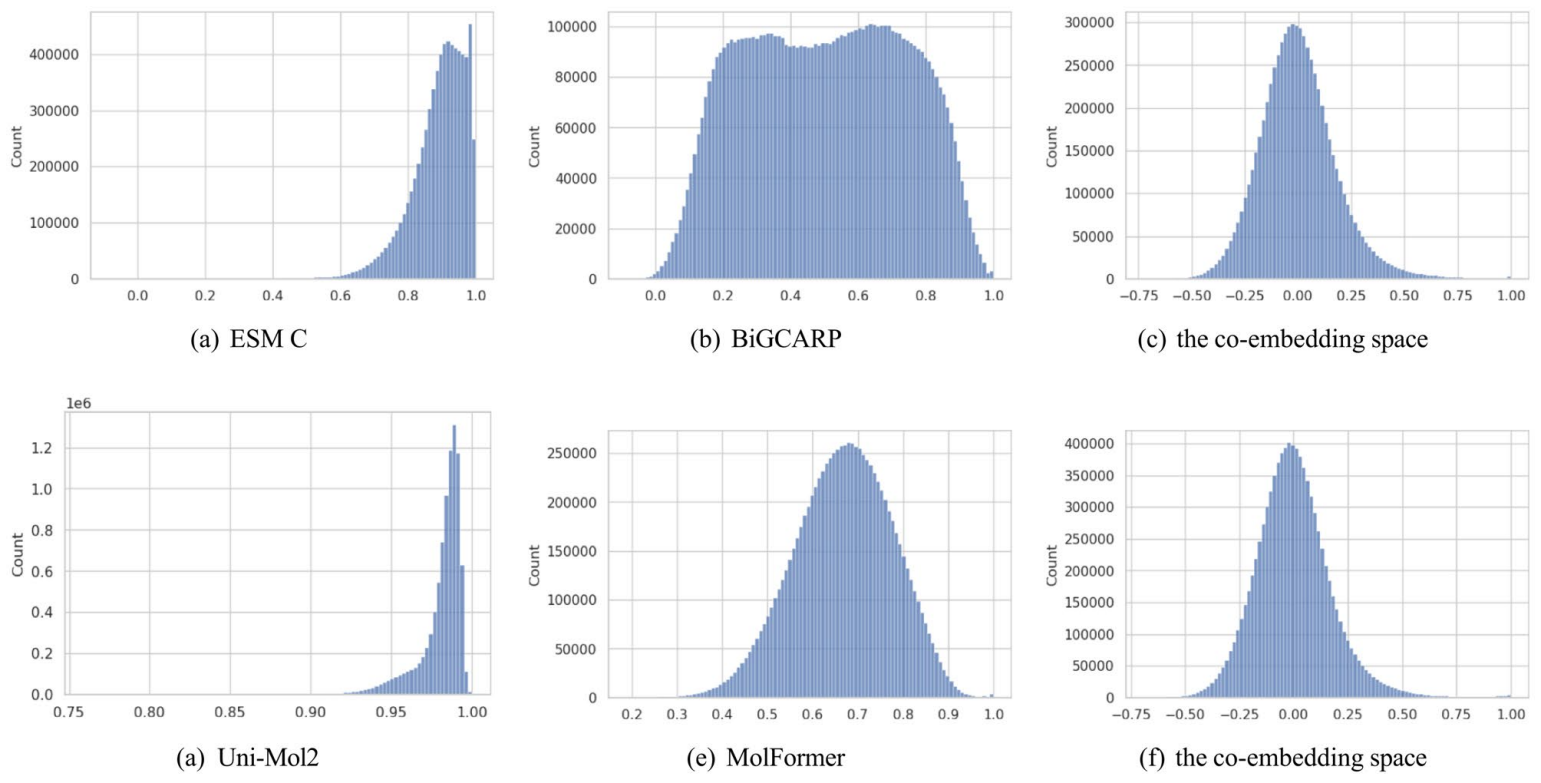
Distribution of similarities over initial embeddings and embeddings in the co-embedding space. Top row: similarities between BGCs; bottom row: similarities between compounds.

### Hyper-parameter tuning

In this subsection, we study the impact of several hyper-parameters, including loss function, sequence length, dimension of co-embedding space, factor multiplied to cosine similarity in Eqs. (4) and (5), on the performance of BCCoE. We show Recall@K under 10-fold cross validation only in Fig. 8. The results under other settings are similar.

Among the three loss functions, N-pair loss performs substantially better than BCE loss and triplet loss especially when K is small. BCCoE is not sensitive to sequence length and embedding dimension. For the factor multiplied to cosine similarity, larger values perform better when K is small and small values perform better when K is large. Considering that top predictions are more important, a value of 5 makes a good trade-off among different values.

The length of the input sequences to the transformer encoder in Fig. 1 is set to 128. If the sequence length of a BGC or a compound is larger than 128, we use the first length-128 sub-sequence only. We tried another strategy which samples length-128 sub-sequences using a sliding window with a gap of 12 (10%) during inference, and then take the max over the samples as the final score. Figure 9 shows that the two strategies perform very closely.

### Performance of models using other foundation models

Besides BiGCARP and MolFormer, other foundation models can also be employed in our framework to generate the initial embeddings of BGCs and compounds. Here we test two other foundation models, ESM C^12^ and Uni-Mol2^16^ to see how different foundation models affect the performance of BCCoE and KNN-2hop. ESM C is a language model trained on protein sequences. We use the 600M-parameter ESM C model to generate initial embedding sequences of BGCs. Uni-Mol2 is a molecular language model integrating atomic level, graph level and geometry structure level features. We use the 1.1B-parameter Uni-Mol2 model to generate the initial embedding sequences of compounds. For both BCCoE and KNN-2hop, when we replace BiGCARP with ESM C, MolFormer is used to generate initial embeddings for compounds; when we replace MolFormer with Uni-Mol2, BiGCARP is used to generate the initial embeddings for BGCs.

The performance of BCCoE is relatively stable when different foundation models are used as shown in Fig. 10. For KNN-2hop, ESM C performs much worse than BiGCARP for retrieving compounds for BGCs (figures on the left) and Uni-Mol2 performs much worse than MolFormer for retrieving BGCs for compound (figures on the right). The reason behind this is that the rankings among compounds/BGCs retrieved by KNN-2hop using one hop or two hops depend on the distribution of the similarities. If the similarities over the first hop is all close to one, then compounds/BGCs retrieved using two hops will mostly be ranked at bottom.

To better understand the behaviors of KNN-2hop, we show the distribution of cosine similarities over initial embeddings and embeddings in the co-embedding space in Fig. 11. Similarities of computed over initial embeddings generated by ESM C and Uni-Mol2 are both highly skewed with most of their values are close to 1, while similarities computed over initial embeddings generated by BiGCARP and MolFormer are more balanced. When ESM C is used and the task is to retrieve compounds for BGCs, similarities over the first hop are similarities between BGCs computed over initial embeddings generated by ESM C and 74.9% of them are above 0.85; similarities over the second hop are similarities between compounds computed over initial embeddings generated by MolFormer, and 96.1% of them are below 0.85. As a result, it is hard for compounds retrieved using two hops to make it to the top. The situation is even more extreme when Uni-Mol2 is used and the task is to retrieve BGCs for compounds. In this setting, similarities over the first hop are similarities between compounds computed over initial embeddings generated by Uni-Mol2 and 99.8% of them are above 0.9; similarities over the second hop are similarities between BGCs computed over initial embeddings generated by BiGCARP and 99.7% of them are below 0.9.

BCCoE does not have the above issue since it performs alignment in the co-embedding space first and then do retrieval in the co-embedding space. Similarities in the co-embedding space exhibit a bell-shaped normal distribution as shown in the last column of Fig. 11. Being stable with respect to initial embeddings generated by different foundation models is another advantage of BCCoE over the no-alignment approach in addition to the higher performance in retrieval.

**Table 6:** New BGC-compound pairs in MIBiG 4.0 that are ranked in top-5 by one model but are ranked beyond top-100 by the other model.

No	Query	Hit	BCCoE		KNN-2hop	
	Compounds	BGCs	Score	Rank	Score	Rank
1	Hassallidin C	BGC0000369	0.581	1	0.851	142
2	Allocyclinone B	BGC0001500	0.716	1	0.868	137
3	Hassallidin D	BGC0000369	0.574	1	0.863	127
4	4-Hydroxy-6-(2-oxoheptadecyl)pyran-2-one	BGC0002332	0.638	2	0.676	1541
5	Isofuranonaphthoquinone A	BGC0001625	0.619	2	0.846	116
6	Livipeptin	BGC0001168	0.606	3	0.669	1797
7	Paeninodin	BGC0001356	0.723	3	0.744	1297
8	Adipostatin A	BGC0002332	0.728	3	0.706	1077
9	Dithioclapurine	BGC0001365	0.532	3	0.760	680
10	Tetrathioclapurine	BGC0001365	0.475	3	0.764	671
11	Trithioclapurine	BGC0001365	0.509	3	0.765	670
12	Reductasporine	BGC0001224	0.620	3	0.733	347
13	10-Oxodehydrodihydrobotrydial	BGC0000631	0.502	3	0.785	162
14	4-Hydroxy-6-pentadecylpyran-2-one	BGC0002332	0.580	4	0.691	1219
15	Ent-kauren-16alpha-ol	BGC0002221	0.831	4	0.822	516
16	Microansamycin F	BGC0001666	0.411	4	0.860	126
17	Microansamycin E	BGC0001666	0.365	5	0.834	226
18	Fluostatin C	BGC0001904	0.710	5	0.842	118
19	Allocyclinone A	BGC0001500	0.652	5	0.837	109
20	Secalonic acid B	BGC0001886	0.274	112	0.913	3

**Table 7:** Routes from the query compound to the retrieved BGCs by KNN-2hop for the 20 cases in Table 6.

No	Query	1st hop		2nd hop	
	Compound	Compound	Score	BGC	Score
1	Hassallidin C	Hassallidin E	0.929	BGC0001614	0.916
2	Allocyclinone B	WS 79089D	0.933	BGC0002732	0.931
3	Hassallidin D	Hassallidin E	0.942	BGC0001614	0.916
4	4-Hydroxy-6-(2-oxoheptadecyl)pyran-2-one	Hierridin B	0.852	BGC0001962	0.793
5	Isofuranonaphthoquinone A	JBIR-77	0.904	BGC0001386	0.936
6	Livipeptin	Intermediate 1	0.872	BGC0002166	0.767
7	Paeninodin	Lariatin	0.921	BGC0000575	0.808
8	Adipostatin A	Hierridin B	0.890	BGC0001962	0.793
9	Dithioclapurine	Aspirochlorine	0.860	BGC0001123	0.884
10	Tetrathioclapurine	Aspirochlorine	0.865	BGC0001123	0.884
11	Trithioclapurine	Aspirochlorine	0.866	BGC0001123	0.884
12	Reductasporine	Erdasporine B	0.868	BGC0001336	0.845
13	10-Oxodehydrodihydrobotrydial	Botcinin K	0.785		
14	4-Hydroxy-6-pentadecylpyran-2-one	Hierridin B	0.872	BGC0001962	0.793
15	Ent-kauren-16alpha-ol	Taxa-4(5),11(12)-diene	0.924	BGC0002394	0.889
16	Microansamycin F	Hygrocin A	0.897	BGC0000075	0.960
17	Microansamycin E	Hygrocin A	0.869	BGC0000075	0.960
18	Fluostatin C	WS79089A	0.917	BGC0001376	0.919
19	Allocyclinone A	WS 79089D	0.900	BGC0002732	0.931
20	Secalonic acid B	Neosartorin	0.951	BGC0001988	0.960

### Top BGCs retrieved by BCCoE and KNN-2hop for new compounds in MIBiG 4.0

In this subsection, we take a closer look at the difference between the predictions made by BCCoE and KNN-2hop. We use both models to retrieve BGCs for new compounds in MIBiG 4.0. BGCs from MIBiG 3.1 are used as the candidate pool. There are 19 ground-truth BGCs that are ranked by BCCoE within top-5 but are ranked by KNN-2hop beyond top-100, while there is only one ground-truth BGC that is ranked by KNN-2hop within top-5 but are ranked by BCCoE beyond top-100. These 20 cases are shown in Table 6. The routes from the query compounds to the BGCs retrieved by KNN-2hop are shown in Table 7. The differences demonstrate that even though KNN-2hop has inferior performance to BCCoE overall, but there are still cases where it can performs better than BCCoE. It may be beneficial to combine both approaches for even better performance. We will explore this in our future work.

**Table 8:** Performance of BGC-MAP, KNN-2hop and BCCoE on retrieving three experimentally validated BGC-compound pairs.

Task	Query	Ground-truth	BGC-MAP		KNN-2hop		BCCoE	
			Rank	Score	Rank	Score	Rank	Score
BGC2Chem	PX048350	dapalide A	1889	0.40	501	0.83	**449**	0.20
	PV754024	lyngbyapeptin A	43	0.76	824	0.83	**5**	0.77
	KY464182	merosterol A	3542	0.23	1697	0.72	**536**	0.42
Chem2BGC	dapalide A	PX048350	395	0.40	728	0.83	**207**	0.20
	lyngbyapeptin A	PV754024	46	0.76	69	0.83	**4**	0.77
	merosterol A	KY464182	803	0.23	1390	0.72	**39**	0.42

For each BGC-compound pair, the highest rank among the three models is in bold.

### Retrieving experimentally validated BGC-compound pairs not in MIBiG

To further demonstrate the practical utility of our multimodal co-embedding framework in real-world natural product discovery, we applied it to three experimentally validated BGC-compounds pairs outside MIBiG database as in BGC-MAP^21^ paper: (PX048350, dapalide A)^23^, (PV754024, lyngbyapeptin A)^24^ and (KY464182, merosterol A)^25^. The same settings are used for the two retrieval tasks as in BGC-MAP^21^ paper: when retrieving BGCs for a given query compound, the candidate BGCs are taken from MIBiG 4.0 dataset plus the three ground-truth BGCs; when retrieving compounds for a given query BGC, the candidate compounds consist of 10826 compounds from Natural Product Atlas (NP Atlas) database, plus the three ground-truth compounds. BCCoE is trained on MIBiG 4.0 using BiGCARP and MoLFormer as foundation models. Table 8 shows the ranks and scores of the three pairs by BGC-MAP, KNN-2hop and BCCoE. The results of BGC-MAP are taken from its original paper and supplementary materials. BCCoE consistently outperforms the other two methods by ranking the three pairs much higher in both tasks, which demonstrates the benefits of using pre-trained foundation models for capturing the information in BGCs Pfam sequences and SMILES and projecting representations of both modalities into a common embedding space.

## Discussion

In this work, we present a generalizable framework for aligning biosynthetic gene clusters (BGCs) and their corresponding natural products within a shared latent space. By integrating large pre-trained language models—BiGCARP for BGC sequences and MoLFormer for chemical structures—into a metric learning paradigm, our approach bridges genomic and chemical modalities through cross-modal representation learning. The resulting co-embedding space captures fine-grained relationships between protein domain architectures and molecular structures, enabling accurate retrieval of BGC-compound pairs and highlighting latent biochemical organization beyond sequence or structure alone.

Critically, the learned representations not only recapitulate known natural product classes but also support bidirectional retrieval tasks central to both traditional and inverse natural product discovery workflows. This dual capability allows for the prioritization of candidate biosynthetic clusters given a target molecule, or the identification of plausible chemical products from newly sequenced strains. As demonstrated across three distinct evaluation settings—including extrapolation to unseen compound classes and forward prediction of novel pairs from future database releases—the model exhibits strong performance and robustness. These results underscore its practical value in reducing the scale of experimental validation in high-throughput screening.

We compared our BGC-Chemical Co-Embedding (BCCoE) model against two non-aligned baseline methods: a standard nearest-neighbor (KNN) approach and a two-hop retrieval strategy (KNN-2hop). While KNN-2hop improves over KNN by incorporating indirect similarity chains, it consistently under-performs relative to BCCoE. Moreover, KNN-2hop exhibits greater sensitivity to the initial embeddings generated by foundation models. As shown in Fig. 10, its performance degrades significantly when the similarity landscape becomes overly uniform—an issue that arises when foundation model embeddings are saturated (i.e. nearly all cosine similarities approach one). In contrast, BCCoE remains stable across diverse initialization conditions, suggesting that the alignment procedure provides meaningful inductive structure and regularization.

The co-embedding space provides a foundation for prioritizing experimental validation in natural product discovery workflows. By grounding BGC selection in a metric space trained on validated BGC-compound associations, BCCoE complements existing annotation tools and biosynthetic expertise to reduce resource-intensive fermentation and screening efforts. Future work may explore integration with generative approaches, though the current model’s primary value lies in its demonstrated ability to enrich candidate lists by 10-100x over random selection for experimentally validated applications.

## Data Availability

The codes of the BCCoE model and the four baseline methods, alongside the datasets generated and/or analysed during the current study are available at https://zenodo.org/records/18849052 .
